# Validity of electron beam computed tomography for coronary artery disease: asystematic review and meta-analysis

**DOI:** 10.1186/1741-7015-5-35

**Published:** 2007-11-25

**Authors:** Nandini Dendukuri, Keith Chiu, James M Brophy

**Affiliations:** 1 Technology Assessment Unit, McGill University Health Center, 687 Pine Avenue West R4.09, Montreal, PQ, H3A 1A1 Canada; 2 Queen Elizabeth Hospital, University Hospital Birmingham NHS Foundation Trust, Birmingham, West Midlands B15 2TH, UK

## Abstract

**Background:**

Electron beam computed tomography (EBCT) is a method for measuring coronary calcification and has been promoted as a possible non-invasive screening/diagnostic tool for coronary artery disease (CAD). Our objective was to carry out a systematic review and meta-analysis of EBCT for the screening of asymptomatic patients and the diagnosis of symptomatic patients for CAD.

**Methods:**

Studies were identified from the PUBMED, MEDLINE, EMBASE, Current Contents, INAHTA and Cochrane Collaboration databases. We identified studies published in English evaluating EBCT using: (1) a prospective design among asymptomatic patients where CAD was measured in terms of clinical outcomes (e.g. myocardial infarction, death, revascularization); and (2)a cross-sectional design among symptomatic patients where CAD was measured by coronary angiography. We compared the risk of CAD in EBCT score categories defined as low (0–10), moderate (11–400) and high (>400). A hierarchical meta-analysis was used to pool risk ratios comparing categories across studies.

**Results:**

We identified 9 studies of asymptomatic patients and 10 studies of symptomatic patients. In both types of studies, we found variability in EBCT category distribution and risk of CAD within categories. For studies of asymptomatic patients we estimated the following risk ratios (95% credible intervals): moderate versus low 3.5 (2.4, 5.1) and high versus low 9.9 (5.3, 17.6). Similar results were obtained for studies of symptomatic patients. Ratios comparing the risk of no CAD among symptomatic patients were as follows: moderate versus low 0.5 (0.3, 0.8) and high versus low 0.12 (0.05, 0.2).

**Conclusion:**

Increasing EBCT scores indicate higher risk for CAD in both asymptomatic and symptomatic patients. In general, asymptomatic patients with EBCT scores in the high category can perhaps be considered for preventive medical therapy and risk factor modification. Symptomatic patients with EBCT scores in the low category can perhaps, at least temporarily, avoid invasive coronary angiography. However, the non-uniform quality of studies and the lack of availability of individual-level data preclude the extension of our results to individual patients.

## Background

Despite tremendous advances in the prevention and treatment of coronary artery disease (CAD), diagnosis and prognosis remain difficult issues. The measurement of coronary calcium deposits has been proposed as a new non-invasive diagnostic tool. Calcium deposition can be quantified non-invasively at a very early stage by electron beam computed tomography (EBCT) using the Agatston method [1]. EBCT scanners are not as versatile as multidetector slice computed tomography (MDCT), but their technological simplicity without moving parts permits more rapid examinations at lower costs [2]. While MDCT is also widely used for the assessment of coronary calcium, the current article focuses on evaluating EBCT. A more detailed examination of EBCT versus MDCT technology appears in a recent scientific statement from the American Heart Association [2].

While the measurement of coronary calcification using EBCT has emerged as a promising screening and diagnostic tool for CAD, there is concern about widespread dissemination of this technology into routine clinical practice before adequate evaluation. Herein we provide a systematic review of the literature on the efficacy of EBCT with separate analyses for both asymptomatic and symptomatic patients. We improve upon previous meta-analyses by: (1) updating previous conclusions with results of recent articles; (2)providing quantitative support for guidelines defining low, moderate and high EBCT scores [3] for both asymptomatic and symptomatic patients; and (3) providing risk ratios for comparing both positive and negative predictive values between low, moderate and high EBCT score categories.

## Methods

### Data sources and searches

We searched the following electronic literature databases: PUBMED, MEDLINE, EMBASE, Current Contents, INAHTA and Cochrane Collaboration. Search terms were 'electron beam tomography' OR 'electron beam' OR 'EBT' OR 'EBCT' OR 'ultrafast' AND 'coronary artery disease' OR 'coronary blood vessel' OR 'coronary' AND 'calcification' OR 'calcium'. Bibliographies of identified articles were searched further. We included studies that were published before 31 July 2006.

### Study selection, data extraction and quality assessment

We required that studies: (1) were published in English; (2) recruited consecutive patients; (3) followed a prospective design for studies of asymptomatic patients; and (4)were designed such that both EBCT and coronary angiography were carried out within 3 months in studies of symptomatic patients. From each study we extracted details of the method of recruitment, inclusion/exclusion criteria, length of follow-up and percentage of completed follow-up (for prospective studies), details of the EBCT protocol, EBCT categories, distribution of patients across EBCT categories, outcome definition, percentage of patients with the outcome of interest in each EBCT category and aggregate results on covariates such as age, sex, smoking, diabetes, hypertension, hypercholesterolemia and history of CAD. Data were extracted by two of the authors (KC and ND). Relevant items from the Standards for Reporting of Diagnostic Accuracy (STARD) guidelines were used to evaluate the quality of individual articles [4].

### Data synthesis and analysis

#### Defining categories on the EBCT scale

There are no standardized EBCT cut-offs, which makes comparative analyses difficult. One guideline [3] has suggested the following cut-offs: 0 (very low), 1–10 (low), 11–100 (moderate), 101–400 (moderately high), >400 (high). For each selected study, we elected to transform the reported categories as closely as possible into one of three standardized categories: low (0–10), moderate (11–400) and high (>400). The lowest and highest categories in a given study were always classified as low and high, respectively. When there was uncertainty about the EBCT classification we carried out sensitivity analyses by placing the category into the adjacent group.

#### Hierarchical meta-analysis comparing predictive values between EBCT categories

For each category we calculated the probability of the outcome (positive predictive value) and the probability of the absence of the outcome (negative predictive value). Given the variability in predictive values across studies, we decided that only the ratios comparing predictive values between categories could reasonably be pooled across studies. This was done by means of a hierarchical meta-analysis [5]. A separate model was fit for each pair of categories that were compared. We chose to compare categories using risk ratios, rather than the commonly used odds ratio, for greater interpretability [5]. Non-informative prior distributions were used for all parameters, thus the results reflect the information in the observed data. We reported the posterior median and 95% credible intervals for the parameters of interest. The models were implemented using WinBUGS software. A copy of the program is available from the first author upon request.

## Results

**Figure 1:**
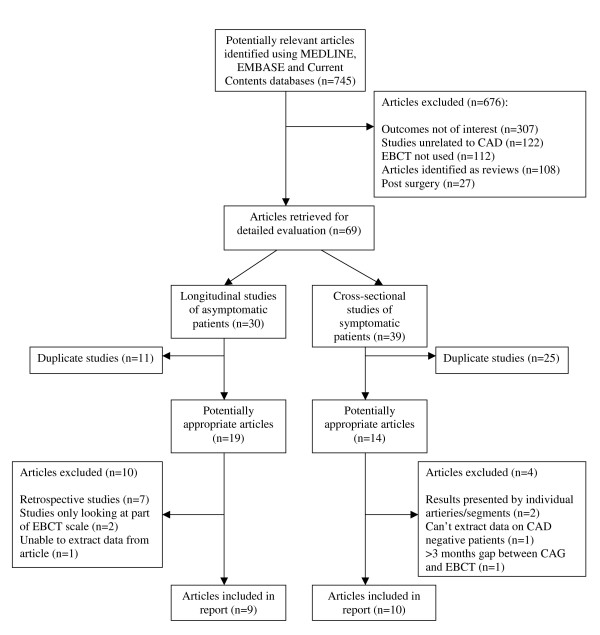
Flowchart summarizing study selection.

The results of the literature search are summarized in Figure 1. From 745 initial studies, we identified 9 prospective studies of asymptomatic patients [6-14] and 10 cross-sectional studies of symptomatic patients [15-24].

### Quality of individual studies

Tables 1 and 2 summarize the quality of each study according to relevant criteria from the STARD guidelines [4]. Most studies of asymptomatic patients excluded patients with a history of heart disease or with a suspected myocardial infarction (MI). However, there was variability in the source population: in some studies patients were self-referred, while in others they were referred by a physician or identified during a routine annual examination. These studies reported the risk of hard outcomes (unstable angina, MI, stroke, coronary death, all-cause mortality) and sometimes also included the softer cardiac outcome of coronary revascularization (Table 3) [14,11]. Most studies of symptomatic patients recruited patients with MI or suspected MI, who would normally be considered candidates for angiography. All of these studies defined coronary disease as the presence of at least 50% coronary stenosis based on angiographic findings. A summary of the demographic and clinical characteristics from the selected studies is given in Table 4. There was no clear difference in the distribution of age, sex, smoking and history of CAD among studies of asymptomatic and symptomatic patients based on the reported aggregate data. The percentage of patients with hypertension and diabetes was somewhat higher in studies of symptomatic patients compared with asymptomatic patients.

**Table 1:** Quality of studies of asymptomatic subjects

**Item**	**Value**	**Wong *et al*. [6]**	**Arad *et al*. [11]**	**Raggi *et al*. [7]**	**Kondos *et al*. [8]**	**Shaw *et al*. [9]**	**Greenland *et al*. [10]**	**Taylor *et al*. [12]**	**Vliegenthart *et al*. [13]**	**LaMonte *et al*. [14]**
Patient recruitment	1: Self referred	2	NR	2	1	2	1	3	3	2
	2: Clinician referred									
	3: Population sample									
Exclusion criteria	1: Did not exclude history of heart disease	2	2	NR	2	2	2	2	2	2
	2: Excluded history of heart disease									
Outcome defined	1: All cause mortality	2	2	3	3	1	3	3	2	2
	2: Coronary outcomes* and revascularization									
	3: Coronary outcomes* only									
EBCT categories	1: Quartiles or pre-selected cut-offs	1	1	2	1	1	1	1	1	1
	2: Age – sex adjusted									
Outcome extraction blinded to EBCT score	1: No	2	2	2	2	2	2	2	2	2
	2: Yes									
Demographic characteristics, risk factors	NR: Not reported	1	1	1	1	1	1	1	1	1
	1: Reported (see Table 3)									
Percentage follow-up	NR: Not reported	NR	1	1	1	1	1	1	1	1
	1: Yes (see Table 4)									
Adjusted results available	1: Yes but cannot be used in meta-analysis	1	1	3	2	3	3	2	3	3
	2: Yes at single cut-off and can be used in meta-analysis.									
	3: Yes at multiple cut-offs and can be used in meta-analysis									
Total	Maximum: 17	11	11	14	13	13	14	15	15	14

*Coronary outcomes: angina, myocardial infarction, coronary death.

**Table 2:** Quality of studies of symptomatic subjects

**Item**	**Value**	**Budoff *et al*. [15]**	**Baumgart *et al*. [16]**	**Seese *et al*. [17]**	**Yao *et al*. [18]**	**Chen *et al*. [19]**	**Bielak *et al*. [20]**	**Hosoi *et al*. [21]**	**Budoff *et al*. [22]**	**Almeda *et al*. [23]**	**Knez *et al*. [24]**
Patient recruitment	NR: Not reported	1	1	1	1	1	1	1	1	NR	1
	1: Undergoing CAG for clinical indications										
Exclusion criteria	NR: Not reported	NR	1	NR	NR	1	1	NR	1	NR	NR
	1: History of CAD/revascularization										
Outcome defined	1: No	2	2	2	2	2	2	2	2	2	2
	2: Yes										
EBCT categories	1: Quartiles or pre-selected cut-offs	1	1	1	1	1	1	1	1	1	1
	2: Age – sex adjusted										
Outcome measurement blinded to EBCT score	1: No	NR	2	NR	NR	2	NR	2	2	NR	2
	2: Yes										
Demographic characteristics	NR: Not reported	1	1	1	NR	1	1	1	1	1	1
	1: Reported										
Risk factors	NR : Not reported	NR	NR	NR	NR	1	1	1	NR	1	1
	1: Reported (see Table 3)										
Adjusted results available	1: Yes but cannot be used in meta-analysis	1	NR	NR	NR	2	3	1	2	2	NR
	2: Yes at single cut-off and can be used in meta-analysis.										
	3: Yes at multiple cut-offs and can be used in meta-analysis										
Total	Maximum: 13	6	8	5	4	11	10	9	10	7	8

**Table 3:** Definition of outcomes and length of follow-up in studies of asymptomatic subjects

**Study**	Definition of outcome*	**Mean length of follow-up (years)**	**Follow-up completed (%)**
1. Wong *et al*. [6]	MI, stroke, revascularization	3.3	---
2. Raggi *et al*. [7]	MI, sudden cardiac death or death due to MI	2.7	100%
3. Kondos *et al*. [8]	Death (owing to CHD or unknown cause), MI	3.1	64%
4. Shaw *et al*. [9]	All-cause mortality	5	100%
5. Greenland *et al*. [10]	MI or CHD death	7.0	87.5%
6. Arad *et al*. [11]	Coronary death, non-fatal MI, CABG, PTCA	4.3	94%
7. Taylor *et al*. [12]	Sudden cardiac death, MI, unstable angina	3.0	99.2%
8. Vliegenthart *et al*. [13]	Incident MI, CHD mortality, revascularization	3.3	~100%
9. LaMonte *et al*. [14]	Non-fatal MI or death from coronary causes, coronary revascularization	3.5	70%

*CABG, coronary artery bypass graft; CHD, coronary heart disease; MI, myocardial infarction; PTCA, percutaneous transluminal coronary angioplasty.

**Table 4:** Summary of demographic characteristics and risk factors

**Study**	**Age mean (SD) (years)**	**Male (%)**	**Smokers (%)**	**Hypertension (%)**	**Diabetes (%)**	History of CAD* (%)
**Studies of asymptomatic subjects**						

1. Wong *et al*. [6]	64 (--)	76	24	26	5	---
2. Raggi *et al*. [7]	52 (9)	50	65	52	15	47
3. Kondos *et al*. [8]	51 (9)	74	48	20	3	---
4. Shaw *et al*. [9]	53 (0.1)	60	40	44	9	69
5. Greenland *et al*. [10]	66 (8)	90	18	41	-	---
6. Arad *et al*. [11]	53 (11)	71	10	34	6	21
7. Taylor *et al*. [12]	43 (3)	82	8	29	1	32
8. Vliegenthart *et al*. [13]	71 (6)	43	16	60	12	19
9. LaMonte *et al*. [14]	54 (10)	64	9	18	3	---

**Studies of symptomatic subjects**						

1. Budoff *et al*. [15]	56 (12)	64	---	---	---	---
2. Baumgart *et al*. [16]	54 (9)	79	---	---	---	---
3. Seese *et al*. [17]	55 (8)	87	---	---	---	---
4. Yao *et al*. [18]	---	---	---	---	---	---
5. Chen *et al*. [19]	66 (10)	85	53	59	16	32
6. Bielak *et al*. [20]	56 (11)	76	62	45	13	51
7. Hosoi *et al*. [21]	63 (-)	67	---	56	36	---
8. Budoff *et al*. [22]	58 (11)	63	---	---	---	---
9. Almeda *et al*. [23]	60	78	14	51	18	57
10. Knez *et al*. [24]	62 (10)	78	23	66	22	---

*CAD, coronary artery disease.

### EBCT protocol

Eighteen studies followed the Agatston method for scoring; only one study followed the Erlanger method for scoring [17]. While all studies used a threshold of greater than 130 Hounsfield units to identify a calcified lesion, there was wide variability in the minimum area in which the signal had to be observed ranging from 0.44 to 1.02 mm^2^. The width of each slice was 3 mm in all studies except one, which used 6 mm slices[10]. The scan time per rotation was 100 ms in all studies. The percentage of the R-R interval to which the scan acquisition trigger was set was typically 80%.

### Results from prospective studies of asymptomatic patients

#### Distribution of EBCT categories and risk of outcome

**Figure 2:**
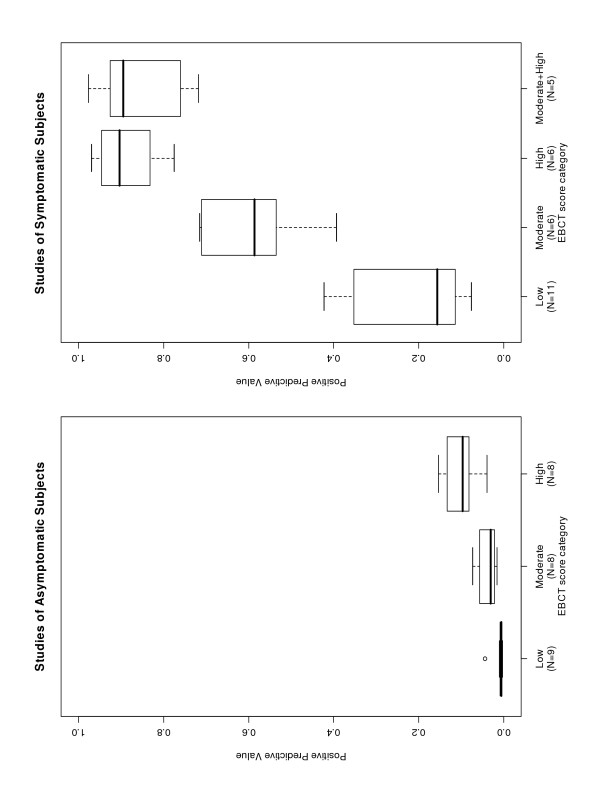
Distribution of positive predictive values across EBCT score categories.

The distributions of EBCT scores as reported in each study are given in Table 5 along with the summary categorization from low to high. The cut-offs reported by individual studies varied greatly, although most studies reported the number of patients with a calcium score of zero. The probability of the outcome increased with increasing EBCT score in all studies (Figure 2 and Table 5). Given the considerable variation between studies in the risk of developing CAD (see Table 5), we concluded that it was not clinically meaningful to pool predictive values across studies. We concentrated instead on pooling the ratios of moderate- or high-risk categories to the baseline low category from each study.

**Table 5:** EBCT score distribution and positive predicted values in studies of asymptomatic patients

	**Reported classification**			**Simplified classification**		
**Study**	**EBCT category**	**Number (%) of patients**	**Number (%) with outcome**	**EBCT Category**	**Number (%) of patients**	**Number (%) with outcome**

1. Wong *et al*. [6]	0	398 (42.9)	4 (1.0)	Low		
	1–15	133 (14.3)	1 (0.8)	Low	531 (57.2)	5 (0.1)
	16–80	134 (14.4)	5 (3.7)	Moderate		
	81–270	131 (14.1)	7 (5.3)	Moderate	265 (28.6)	12 (4.5)
	≥271	132 (14.2)	11 (8.3)	High	132 (14.2)	11 (8.3)
	*Total*	*928*	*28*			
2. Raggi *et al*. [7]	0	292 (46.2)	1 (0.3)	Low	292 (46.2)	1 (0.3)
	1–99	219 (34.7)	12 (5.5)	Moderate		
	100–400	74 (11.7)	8 (10.8)	Moderate	293 (46.3)	20 (6.8)
	>400	47 (7.4)	6 (12.8)	High	47 (7.4)	6 (7.4)
	*Total*	*632*	*27*			
3. Kondos *et al*. [8]	0	1816 (32.2)	5 (0.3)	Low	1816 (32.2)	5 (0.3)
	>0	3819 (67.8)	53 (1.4)	Moderate/high	3819 (67.8)	53 (1.4)
	*Total*	*5635*	*58*			
4. Shaw *et al*. [9]	≤10	5946 (57.3)	62 (1.0)	Low	5946 (57.3)	62 (1.0)
	11–100	2044 (19.7)	53 (3.0)	Moderate		
	101–400	1432 (13.8)	54 (3.8)	Moderate	3476 (33.5)	107 (2.9)
	401–1000	623 (6.0)	39 (6.3)	High		
	>1000	332 (3.2)	41 (12.4)	High	955 (9.2)	80 (8.4)
	*Total*	*10377*	*249*			
5. Greenland *et al*. [10]	0	316 (30.7)	14 (4.4)	Low	316 (30.7)	14 (4.4)
	1–100	321 (31.2)	21 (6.5)	Moderate		
	101–300	171 (16.6)	15 (8.8)	Moderate	492 (47.8)	36 (7.3)
	≥301	221 (21.5)	34 (15.4)	High	221 (21.5)	34 (15.4)
	*Total*	*1029*	*84*			
6. Arad *et al*. [11]	0	1504 (25)	8 (0.5)	Low	1504 (25.0)	8 (0.5)
	1–99	1973 (25)	20 (1)	Moderate		
	100–399	686 (25)	38 (5.5)	Moderate	2659 (50.0)	58 (2.2)
	≥400	450 (25)	63 (14)	High	450 (25.0)	63 (14.0)
	*Total*	*4613*	*129*			
7. Taylor *et al*. [12]	0	1261 (77.6)	7 (0.6)	Low	1381 (85.0)	7 (0.6)
	1–9	120 (7.4)	0 (0.0)	Moderate		
	10–44	120 (7.4)	2 (1.7)	Moderate	120 (7.4)	2 (1.7)
	>44	124 (7.6)	5 (4.0)	Moderate/high	124 (7.6)	5 (4.0)
	*Total*	*1625*	*14*			
8. Vliegenthart *et al*. [13]	0–100	905 (50.4)	7 (0.8)	Low	905 (50.4)	7 (0.8)
	101–400	425 (23.7)	13 (3.1)	Moderate	425 (23.7)	13 (3.1)
	401–1000	269 (15.0)	13 (4.5)	High		
	>1000	196 (10.9)	17 (8.7)	High	365 (25.9)	30 (8.2)
	*Total*	*1795*	*50*			
9. LaMonte *et al*. [14]	0	5472 (50.9)	15 (0.3)	Low	5472 (50.9)	15 (0.3)
	1–38 (Men), 1–16 (Women)	1760 (16.4)	19 (1.1)	Moderate		
	39–249 (Men), 17–112 (Women)	1758 (16.4)	62 (3.5)	Moderate	3518 (32.8)	81 (2.3)
	≥250(Men), ≥113 (Women)	1756 (16.3)	191 (10.9)	High	1756 (16.3)	191 (10.9)
	*Total*	*10746*	*287*			

*CAD, coronary artery disease.

#### Meta-analysis

**Figure 3:**
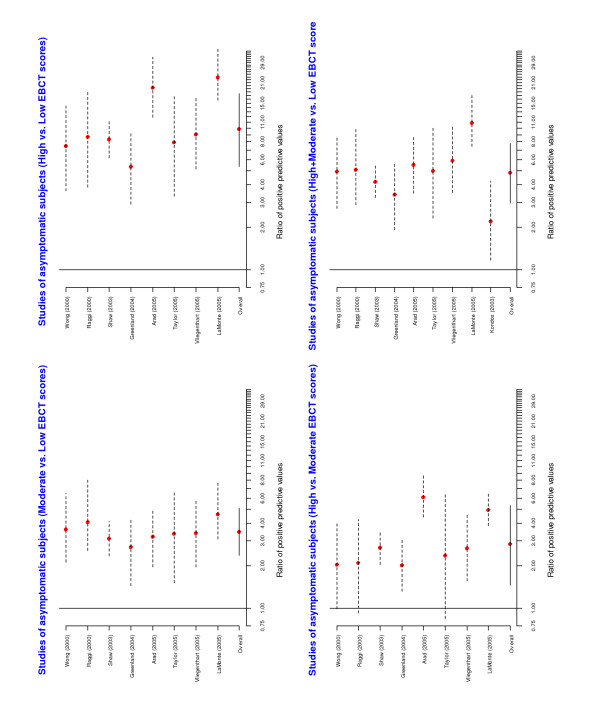
Forest plots from meta-analyses of ratios of positive predictive values among asymptomatic subjects.

**Figure 4:**
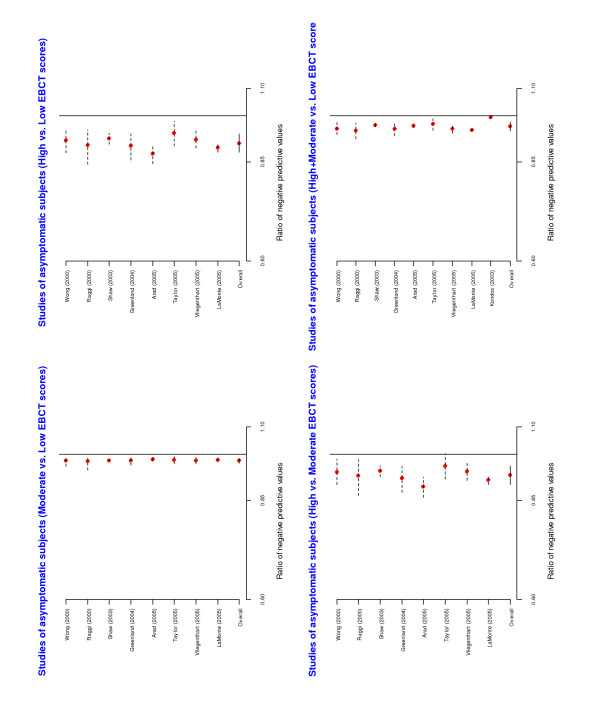
Forest plots from meta-analyses of ratios of negative predictive values among asymptomatic subjects.

A forest plot of the individual and overall risk ratios is given in Figure 3. The pooled risk ratios comparing positive predictive values across EBCT categories were statistically significant. A forest plot of the risk ratios of the negative predicted values is given in Figure 4. The overall risk ratios comparing the three categories were all close to 1, indicating that despite the fact that the negative predictive values were high the EBCT categorization was not very useful for identifying patients unlikely to have CAD.

### Results from cross-sectional studies of symptomatic patients

#### Distribution of EBCT categories and risk of outcome

The distribution of EBCT scores in each study is given in Table 6 along with the summary categorization from low to high. As in the case of studies of asymptomatic patients, there was variation in the reported cut-offs, the distribution of EBCT scores and the risk of CAD in each EBCT category. The negative predictive value (probability of no CAD) corresponding to a calcium score of zero ranged from 0.58 to 0.92 in the individual studies.

**Table 6:** EBCT score distribution and positive predictive values in studies of symptomatic subjects

	**Reported classification**			**Simplified classification**		
	
**Study**	**EBCT category**	**Number (%) of patients**	**Number (%) with CAD**	**EBCT category**	**Number (%) of patients**	**Number (%) with CAD**
Budoff *et al*. [15]	0	147 (20.7)	23 (15.6)	Low	147 (20.7)	23 (15.6)
	>0	563 (79.3)	404 (71.8)	Moderate/high	563 (79.3)	404 (71.8)
	*Total*	*710*	*427*			
Baumgart *et al*. [16]	0	32 (56.1)	10 (31.3)	Low	32 (56.1)	10 (31.3)
	>0	25 (43.9)	19 (76.0)	Moderate/high	25 (43.9)	19 (76.0)
	*Total*	*57*	*29*			
Seese *et al*. [17]	0	22 (20.6)	4 (18.2)	Low	22 (20.6)	4 (18.2)
	>0	85 (79.4)	83 (97.7)	Moderate/high	85 (79.4)	83 (97.7)
	*Total*	*107*	*87*			
Yao *et al*. [18]	0	26 (40.1)	11 (42.3)	Low	26 (40.1)	11 (42.3)
	>0	38 (59.9)	34 (89.5)	Moderate/high	38 (59.9)	34 (89.5)
	*Total*	*64*	*45*			
Bielak *et al*. [20]	0	40 (18.8)	1 (2.5)	Low		
	1–9	31 (14.6)	8 (25.8)	Low	71 (33.3)	9 (12.7)
	10–49	25 (11.7)	6 (24.0)	Moderate		
	50–99	13 (6.1)	7 (53.8)	Moderate		
	100–199	24 (11.3)	17 (70.8)	Moderate		
	200–499	25 (11.7)	21 (84.0)	Moderate?	87 (40.8)	51 (58.6)
	≥500	55 (25.8)	52 (94.5)	High	55 (25.8)	52 (94.5)
	*Total*	*213*	*112*			
Chen *et al*. [19]	0–5	22 (19.0)	2 (9.1)	Low	22 (19.0)	2 (9.1)
	6–75	17 (14.7)	3 (17.7)	Moderate		
	76–500	29 (15.0)	24 (82.8)	Moderate?	46 (29.7)	27 (58.7)
	>500	48 (41.4)	45 (93.8)	High	48 (41.4)	45 (93.8)
	*Total*	*116*	*74*			
Hosoi *et al*. [21]	0	36 (12.8)	7 (19.4)	Low	74 (26.3)	29 (39.2)
	1–10	38 (13.5)	22 (57.9)	Low		
	11–100	59 (20.9)	39 (66.1)	Moderate		
	101–399	53 (18.8)	41 (77.4)	Moderate	112 (39.7)	80 (71.4)
	>399	96 (34)	93 (96.9)	High	96 (34)	93 (96.9)
	*Total*	*282*	*202*			
Budoff *et al*. [22]	0	386 (20.9)	39 (10.1)	Low	386 (20.9)	39 (10.1)
	1–20	216 (11.7)	60 (27.8)	Moderate?		
	21–80	230 (12.4)	108 (47.0)	Moderate		
	81–100	55 (3.0)	29 (52.7)	Moderate	501 (27.1)	197 (39.3)
	>100	964 (52.1)	747 (77.5)	High	964 (52.1)	747 (77.5)
	*Total*	*1851*	*983*			
Almeda *et al*. [23]	0	26 (10.6)	2 (7.7)	Low	26 (10.6)	2 (7.7)
	<100	28 (11.4)	14 (50.0)	Moderate		
	100–399	71 (28.9)	39 (54.9)	Moderate	99 (40.3)	53 (53.5)
	≥400	121 (49.2)	105 (86.8)	High	121 (49.2)	105 (86.8)
	*Total*	*246*	*160*			
Knez *et al*. [24]	0	254 (12.0)	13 (5.1)	Low		
	1–9	427 (20.2)	75 (17.6)	Low	681 (32.2)	88 (12.9)
	10–99	211 (10.0)	150 (71.1)	Moderate	211 (10.0)	150 (71.1)
	>99	1223 (57.8)	1017 (83.2)	High	1223 (57.8)	1017 (83.2)
	*Total*	*2115*	*1255*			

*CAD, coronary artery disease; EBCT, electron beam computed tomography.

**Here '?' denotes that the simplified EBCT category could be the adjacent category.

#### Meta-analysis

**Figure 5:**
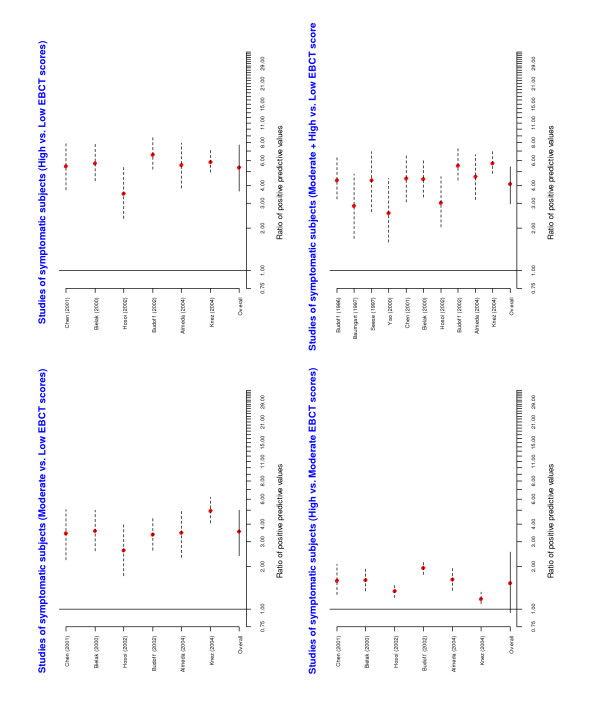
Forest plots from meta-analyses of ratios of positive predictive values among symptomatic subjects.

**Figure 6:**
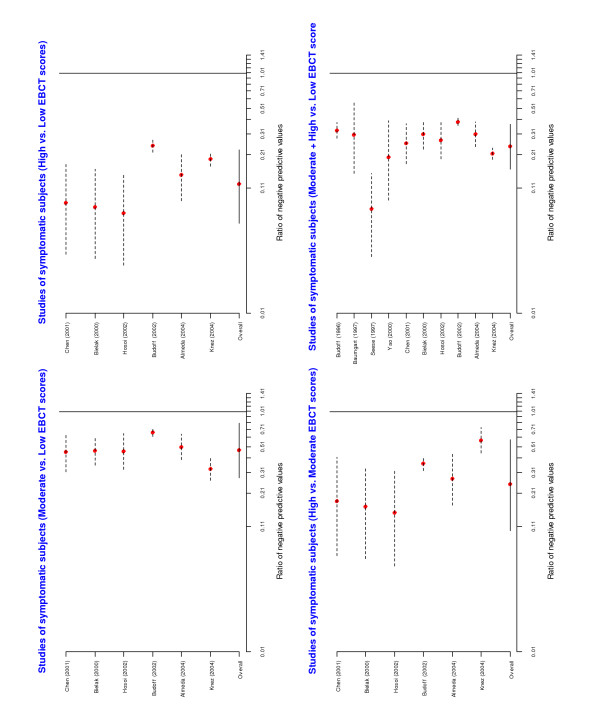
Forest plots from meta-analyses of ratios of negative predictive values among symptomatic subjects.

The results of the meta-analyses are presented in Figures 5 and 6. Once again there was a statistically significant difference in the positive predictive values in the low category compared with the other two categories. In particular, the pooled risk ratios suggest that a symptomatic subject with a low score has approximately one-quarter of the risk of having significant angiographic coronary stenosis than the moderate/high categories. This would mean that if the average patient in the moderate/high category had an 80% risk of CAD, the average patient in the low category would have a 20% risk of CAD. The overall risk ratios comparing negative predictive values were significantly different from 1. In particular, negative predictive values in the low category were much higher than in the other two categories suggesting that a symptomatic subject with a low score has a very small likelihood of having coronary stenosis. For three studies [19,22] there was ambiguity in determining the simplified classification of the EBCT score category. However, repeating the meta-analysis following a reclassification of these categories did not affect our final results.

## Discussion

We have carried out a systematic review and meta-analysis of the published literature on the screening and diagnosis of CAD using EBCT. We identified two different types of studies: (1) 9 prospective studies of asymptomatic patients evaluating the predictive validity of EBCT for a mixture of hard outcomes (unstable angina, MI, stroke, coronary death, all-cause mortality) and revascularization; and (2) 10 cross-sectional studies of symptomatic patients evaluating the concurrent validity of EBCT for coronary stenosis as measured by coronary angiography. The risk of coronary events or angiographically confirmed CAD increased consistently with increasing EBCT scores in both types of studies.

Our results provide quantitative support for the cut-offs proposed by Rumberger *et al*. [3] to determine low-, moderate- and high-risk categories of EBCT scores. These cut-offs have since been used by a number of studies for reporting the validity of EBCT [9,11,13,21,23]. Similar cut-offs have been used in two earlier meta-analyses of studies of asymptomatic patients, including the recently published ACCF/AHA Clinical Expert Consensus Document [25,26]. These cut-offs were determined based on a qualitative review of the literature. For example, an EBCT score of 0 was assumed to be associated with a risk of 5% or less of CAD, while a score of 400 or more was assumed to be associated with a risk of 90% or more of CAD. Based on the studies we have identified, these assumptions seem to be reasonable estimates of the risk of coronary stenosis in symptomatic patients, but not of prognostic risk in asymptomatic patients. Furthermore, we have found that even among studies of symptomatic patients there is considerable variability in these values. Nonetheless, our analyses have shown that the cut-offs proposed by Rumberger *et al*. [3] are clinically meaningful for classifying the aggregate patient into categories associated with a monotonically increasing risk of an adverse cardiac outcome. For asymptomatic patients this difference in risk is statistically significantly different between all categories, while in symptomatic patients the difference between moderate and high categories is only of borderline statistical significance. We found that among asymptomatic patients, negative predictive values in these same categories were very high independent of the category threshold, and thus did not help us to distinguish between categories. Among symptomatic patients, where the disease prevalence is naturally much higher, negative predictive values were lower. However, they were significantly higher in the low EBCT category compared with moderate or high categories.

We cannot comment on the utility of EBCT scores for individual risk stratification as our results were not adjusted for age, sex and other known risk factors. Therefore, while the proposed cut-offs [3] may be useful for making conclusions about the average patient they should not be used for individual risk stratification in light of our knowledge of the age-sex variation in coronary calcium scores [27]. For the average patient, our results suggest that an asymptomatic patient with a high EBCT score may benefit from extensive follow-up possibly supported by intensive medical therapy. Similar conclusions apply to asymptomatic patients classified as having a moderate EBCT score. In symptomatic patients, it appears that the average patient with a low EBCT score may have only a small chance of having coronary stenosis compared with those with high or moderate scores. Thus, they could be further evaluated with non-invasive tests possibly avoiding angiography.

Compared with earlier meta-analyses of asymptomatic subjects [26,28,25], we found four new articles [8,13,14,12] and cohorts with longer follow-up for three previously included articles [10,9,11]. A previous meta-analysis [25] extrapolated data for individual studies in order to make them comparable when the reported EBCT cut-offs were different from the values identified to define low, moderate and high categories. Instead, we chose to use categories that were actually reported and classified them as closely as possible into low, moderate and high. Also, in contrast to this earlier meta-analysis [25], we chose to combine the ratios of predictive values that had not been adjusted for CAD risk factors in order to permit a larger sample size for analysis. Although our ratios of positive predictive values are slightly higher than the values from this meta-analysis [25], they lead to similar conclusions. Similar results were also obtained in a meta-analysis of six studies of asymptomatic patients published recently in the ACCF/AHA Clinical Expert Consensus Document [26]. Compared with the earlier meta-analysis of symptomatic subjects [29], we have identified one additional article [17] and added additional information in two studies [22,1]. Unlike the previous analysis, we treated the results from one multi-centre trial as coming from a single study [15]. Furthermore, we divided the 'calcium positive' scores into moderate and high categories. In our analyses of both types of studies we reported ratios comparing negative predictive values in addition to those comparing positive predictive values.

The large number of potentially relevant studies identified by our literature search reduced to 19 unique studies providing quantitative information that could be used to evaluate the utility of EBCT. Thus, despite the large number of publications about this technology, there is clearly a paucity of useful information for evaluating it. The main limitation of our study relates to the quality of the original publications and their deficiencies in following definitive standards for diagnostic publications. Combining information across the identified studies proved challenging given the lack of a standardized approach for reporting EBCT scores and the variability in recruitment methods/population across studies. Very few studies met more than 80% of the criteria identified by the STARD guidelines for measuring quality of reporting in diagnostic studies.

We were unable to separate results based on age or gender, both of which have been shown to have an important impact on the interpretation of EBCT scores. We were also unable to estimate the incremental value of EBCT beyond established risk factors. Both of these limitations were the result of the lack of access to individual-level data. For studies of asymptomatic patients we were unable to evaluate the change in risk over time in each category, as information on the time of occurrence of outcomes was not always available. Consequently, we were limited to a crude cross-sectional type analysis based only on the observed outcomes in each category at the end of each study. Also incomplete study follow-up may have also introduced bias. Finally, another possible limitation of our work may relate to our literature search where unpublished, grey literature and non-English articles were not considered. On the other hand, our search of the conventional electronic databases may be seen as thorough and systematic.

## Conclusion

We conclude that increasing calcium scores are associated with increasing risk of CAD among both asymptomatic and symptomatic patients. In general, it appears that asymptomatic patients with a high EBCT score may benefit from preventive interventions such as medical therapy and risk factor modification. Similar conclusions apply to asymptomatic patients classified as having a moderate EBCT score. Among symptomatic patients, those with a low EBCT score could perhaps be further evaluated with non-invasive tests possibly avoiding angiography. However, the evidence in the literature does not allow us to draw conclusions about the value of this technology for individual patients, and therefore to justify its routine use. It is especially unclear what additional value EBCT scores provide to patients in different age-sex groups. We recommend that future studies of EBCT need to: (1) use standardized cut-offs to allow for comparability; (2) adjust for the age-sex distribution of EBCT scores in classifying individuals into risk categories; and (3) use survival analysis techniques while reporting data from prospective studies.

## Abbreviations

AHA : American Heart Association; 

CAD : Coronary artery disease; 

CABG : Coronary artery bypass graft; 

CHD : Coronary heart disease; 

EBCT : Electron beam computed tomography;

MI : Myocardial infarction;

NR : Not reported;

PTCA : Percutaneous transluminal coronary angiography;    

STARD : Standards for Reporting of Diagnostic Accuracy.

## Authors' contributions

ND participated in the study design, literature search, extraction of data, data analysis, interpretation of results and writing of the manuscript. KC participated in the literature search, extraction of data and writing of the manuscript. JMB participated in the study design, interpretation of results and writing of the manuscript. All authors read and approved the final manuscript.

## Pre-publication history

The pre-publication history for this paper can be accessed here:


